# Electroenzymatic CO_2_ Fixation

**DOI:** 10.1002/anie.202522125

**Published:** 2026-05-25

**Authors:** Leonardo Castañeda‐Losada, Michael Richter, Christophe Léger, Vincent Fourmond, Nicolas Plumeré

**Affiliations:** ^1^ Fraunhofer Institute for Interfacial Engineering and Biotechnology IGB Bio‐, Chemo‐ and Electrocatalysis BioCat Straubing Germany; ^2^ CNRS, Aix Marseille Univ, BIP Marseille France; ^3^ Campus Straubing for Biotechnology and Sustainability Technical University of Munich (TUM) Straubing Bayern Germany

**Keywords:** aerobic stability, catalytic reversibility, cofactor regeneration, confinement, electron mediation

## Abstract

Replacing fossil resources with renewable alternatives for chemical production requires highly integrated, energy‐efficient, and selective CO_2_ utilization processes. Electroenzymatic CO_2_ fixation offers a promising pathway, combining high energy efficiency with unparalleled product selectivity, making it a viable approach for synthesizing complex molecules. This review outlines the fundamental principles of bioelectrocatalytic CO_2_ conversion using reductases and carboxylases, providing an overview of the available enzymes, their product range, and key thermodynamic and kinetic considerations. By highlighting both the potential and limitations of electrochemical CO_2_ fixation, this review aims to inform future progress in the discovery, engineering, and application of CO_2_‐converting enzymes, with the ultimate goal of realizing the full potential of electroenzymatic CO_2_ fixation for the sustainable synthesis of fine and specialty chemicals.

## Graphical abstract

Electroenzymatic CO_2_ fixation enables energy‐efficient, highly selective synthesis of complex molecules. Unlocking its full potential requires fundamental understanding of electrode‐coupled reductases and carboxylases. This review critically discusses available enzymes, product scope, and key thermodynamic and kinetic considerations, highlighting current opportunities and limitations in electroenzymatic CO_2_ fixation.

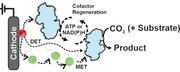

## 1 Introduction

The transition to carbon neutrality and a circular carbon economy will require the replacement of fossil resources with renewable energy and CO_2_ as feedstock for generating chemicals and fuels. Electrocatalytic CO_2_ reduction is progressing toward industrial deployment for producing C_1_ and C_2_ chemicals due to the declining cost and increasing availability of renewable electricity. This process efficiently utilizes electrons from a cathode as reducing equivalents, achieving high atom economy in CO_2_ reduction. Industrially relevant performance metrics have already been achieved for formic acid production with current densities up to 450 mA cm^−2^ and faradaic efficiencies up to 97% [1, 2]. Several other CO_2_‐derived products, including carbon monoxide, ethanol, methanol, and ethylene, are considered economically viable, as their projected minimum selling prices are competitive with current market rates [3, 4]. However, major challenges remain: high overpotentials lead to energy losses of up to 50% for many electrocatalytic CO_2_ reduction reactions. Competitive side reactions, particularly the hydrogen evolution reaction, further reduce efficiency. Combined, these factors result in low energy efficiency in converting electricity into chemical bonds, preventing the economic viability of other products that could otherwise be accessible. Furthermore, the limited selectivity of electrocatalysis restricts the product scope to small molecules.

Biocatalysis in the context of gas fermentation offers an alternative well established approach for CO_2_ utilization [5], particularly suited for the synthesis of complex molecules. The enzymes involved in CO_2_ fixation catalyze multi‐electron, multi‐proton transfer steps with exceptional chemo‐, regio‐, and stereo‐selectivity, enabling carbon chain extension beyond the performance of synthetic materials used in electrocatalysis. In nature, CO_2_ reduction is mainly driven by reducing equivalents generated from water and light through photosynthesis, though the efficiency of light to chemical energy conversion remains low (below 5% [6]). For technological applications, fermentative CO_2_ fixation is currently preferred. However, gas fermentation relies on chemical reducing agents, which are typically sourced from fossil‐derived feedstocks (typically H_2_ or CO). While the enzymes responsible for catalysis of CO_2_ fixation are highly energy efficient, the overall fermentative processes often are not, as energy losses occur due to the cell metabolism [7]. Additionally, energy‐intensive downstream processing of the fermentation products often further inflates costs [8].

**FIGURE 1:**
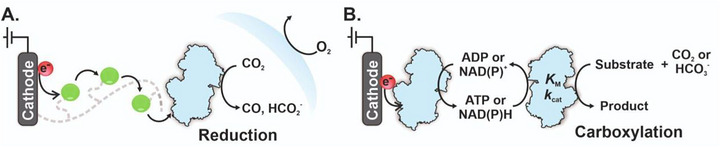
Electroenzymatic CO_2_ fixation via reductases (A) and carboxylases (B). These systems may involve direct or mediated electron transfer, cofactor regeneration, and strategies to protect the enzyme from oxygen. The substrate of carboxylases can be either CO_2_ or carbonate depending on the enzyme. Enzymes are depicted in blue.

To address these limitations, bioelectrocatalytic systems that couple electrochemistry with biocatalysis have been developed, enabling the use of renewable electricity to achieve highly selective CO_2_ conversion with high energy efficiency. While bioelectrocatalysis based on whole cells interfaced with electrodes has reached relatively high technology readiness levels [7], we focus here on the electroenzymatic approach, which utilizes isolated enzymes (reductases and carboxylases) coupled to electrodes (Figure 1). The electroenzymatic strategy enables the wiring of high loading of the biocatalysts on electrodes leading to high current densities, while bypassing energy losses associated with cellular metabolism. Despite its promise, electroenzymatic CO_2_‐fixation remains at a significantly lower technology readiness level than electrocatalysis or whole‐cell bioelectrocatalysis. Advancing this technology requires addressing fundamental challenges, including:

1. Expanding the repertoire of CO_2_‐converting enzymes.
2. Understanding thermodynamic and kinetic constraints.
3. Achieving efficient electron supply and cofactor regeneration.
4. Managing intermediates in multistep transformations.
5. Leveraging enzyme confinement on electrodes.
6. Increasing enzyme stability in (aerobic) electrochemical systems.

This review explores these critical aspects, examining enzyme‐electrode connections (Section 3.1), cofactor requirements (Section 3.2), strategies for high current density (Section 3.3), interplay between kinetics and thermodynamics (Section 3.4), and O_2_ stability (Section 3.5). To support this analysis, we provide two tables summarizing the diversity of CO_2_‐fixing enzymes, detailing their reactions, and their kinetic (Table 1) and thermodynamic properties (Table 2). Where possible, we completed our analysis with the calculation of total turnover numbers (TTN), turnover frequencies (TOF), reaction rates, and *k*_cat_/*K*_M_ values using data from the original references to enable meaningful comparison of the performances across different systems for electroenzymatic CO_2_ fixation. By highlighting the challenges in the context of the diversity of CO_2_‐fixing enzymes, we aim to guide the future development of electroenzymatic CO_2_ fixation as a scalable and sustainable solution for the production of chemicals.

**TABLE 1:** Summary of the substrate, cofactors, and kinetic properties of the most common CO_2_ utilizing enzymes.

#	Carboxylase/Reductase	EC Number	Subs.**^a^**	Cofactor	Red. Eq.**^b^**	*K*_M_^CO2^ (mM)	Spec. activity (µmol min^−1^ mg^−1^)	*k*_cat_ (s^−1^)	O_2_ sen. **^c^** [9]
1	RuBisCO	4.1.1.39	CO_2_	Mg^2+^	Sub.	0.01–0.3 [10], 0.01–0.02 [9]	3–10 [10], 0.9–5 [9]	3–12 [10]	No^e^
2	Phosphoenol pyruvate carboxylase (PEPC)	4.1.1.31	HCO_3_^−^	Mg^2+^, (ATP)	Sub.	0.01–0.03 [10], 0.06–0.2 [9]	20–150 [10], 11–52 [9]	40–150 [10]	No
3	Acetyl‐CoA carboxylase	6.4.1.2	HCO_3_^−^	Mg^2+^, ATP, biotin	Sub.	0.03–0.6 [10], 0.7–1.7 [9]	3–20 [10], 0.5–52 [9]	13–43 [10]	No
4	Propionyl‐CoA carboxylase	6.4.1.3	HCO_3_^−^	Mg^2+^, ATP, biotin	Sub.	0.03–0.6 [10], 0.7–1.7 [9]	3–20 [10], 1.3–62 [9]	13–40 [10]	No
5	Pyruvate carboxylase	6.4.1.1	HCO_3_^−^ [11]	Mg^2+^, ATP, biotin	Sub.	0.03–0.2 [10], 0.25–2.5 [9]	20–30 [10], 3.4–43 [9]	40–90 [10]	No
6	Methyl crotonyl‐CoA carboxylase	6.4.1.4	HCO_3_^−^	Mg^2+^, ATP, biotin	Sub.	0.03–0.2 [10], 0.8–0.9 [9]	3–5 [10], 0.8–9.5 [9]	6–10 [10]	No
7	Acetone carboxylase	6.4.1.6	HCO_3_^−^ (CO_2_)^d^	Mn^2+^/Zn^2+^, ATP	Sub.	2–4 × 10^−6^ [9]	0.2–0.5 [9]		No
8	Urea carboxylase	6.3.4.6	HCO_3_^−^	Mg^2+^, ATP, biotin	Sub.			2–12 [12], 1–210 [13]	No
9	Glycine cleavage system		CO_2_	NADH, PLP, lipoate	NADH				No
10	Phospho gluconate dehydrogenase	1.1.1.44	CO_2_	NADPH	NADPH	15–50 [9]	0.15–1.0 [9]		No
11	Isocitrate dehydrogenase (IDH)	1.1.1.41, 42	CO_2_	NAD(P)H	NADPH	0.02–8 [10], 1.7–3.7 (HCO_3_^−^) [9]	2–38 [10], 3–70 [9]	1–100 [10]	No
12	Crotonyl‐CoA carboxylase/reductase (Ccr)	1.3.1.85	CO_2_	NADPH	NADPH	0.01–0.2 [10], 14 (HCO_3_^−^) [9]	13–100 [10], 1.8–130 [9]	10–80 [10]	No
13	L‐malate: NADP^+^ oxidoreductase	1.1.1.3, 39, 40	CO_2_	NADPH	NADPH	1–5 [10], 3–13 (HCO_3_^−^) [9]	30–70 [10], 0.4–33 [9]	20–280 [10]	
14	Formate dehydrogenase (FDH non‐metal)	1.17.1.9	CO_2_	NADH	NADH	0.01–40 [14], 0.4–30 [15]		0.01–11 [14], 0.008–0.32 [15]	No
15	Formate dehydrogenase (FDH metal Mo‐W)	1.17.1.9	CO_2_	Mo or W	Fd.	0.02–8 [14]		280 [16], 0.01–2654 [14]	Yes
16	Carbon monoxide dehydrogenase (CODH)	1.2.7.4	CO_2_	NiFe_4_S_4_	Fd.	0.3 [17]	0.14–0.3 [17]	45 [18, 19], 420 [20]	Yes
17	2‐ketoglutarate:ferredoxin oxidoreductase (2KFOR)	1.2.7.3	CO_2_	Fd, TPP, CoA	Fd.		< 1 [9]		Yes
18	Pyruvate:ferredoxin oxidoreductase (PFOR)	1.2.7.1	CO_2_	Fd, TPP, CoA	Fd.	3.5 (HCO_3_^−^), 2–5 (CO_2_) [9]	< 1 [9]		Yes

Abbreviations: CoA, ester hydrolyzed during carboxylation; Fd, ferredoxin; Sub., substrate; TPP, thiamine pyrophosphate.

^a^ substrate,

^b^ reducing equivalents,

^c^ O_2_ sensitivity,

^d^ not experimentally validated,

^e^ side reactivity: oxygenation of ribulose‐1,5‐bisphosphate [21].

**TABLE 2:** Thermodynamics of the most common enzymatic CO_2_ fixation reactions.

	Carboxylase	Reaction	∆_r_ *G*° (kJ/mol)		*E* ^0'^ (V vs. SHE)	Bi‐ direc.^b^	Bias ^c^
			Lit.	Calc.^a^			
1	RuBisCO	Ribulose‐1,5‐bisphosphate + CO_2_ → 2 glycerate‐3‐phosphate	−32 [22]	−26.4 ± 7.5		No [9]	Car [9]
2	Phosphoenol pyruvate carboxylase (PEPC)	PEP + CO_2_ → oxaloacetate + Pi	−40 [23], −32.2 [22]	−37.7 ± 6.1		No [9]	Car [9]
3	Acetyl‐CoA carboxylase	Acetyl‐CoA + CO_2_ + ATP → Malonyl‐CoA + ADP + Pi	−9.1 [22], −5.4 [24], −10 [23]	−6.8 ± 2.9		No^f^	Car [9]
4	Propionyl‐CoA carboxylase	Propionyl‐CoA + CO_2_ + ATP → Methylmalonyl‐CoA + ADP + Pi	−9.1 [22], −3.6 [24], −10 [23]	−2.7 ± 10.3		No^f^	Car [9]
5	Pyruvate carboxylase	Pyruvate + CO_2_ + ATP → Oxaloacetate + ADP + Pi		−4.9 ± 1.4		No^f^	Car [9]
6	Methylcrotonyl‐CoA carboxylase	3‐methylcrotonyl‐CoA + CO_2_ + ATP → 3‐methylglutaconyl‐CoA + ADP + Pi		−11.3 ± 2.9		No^f^	Car [9]
7	Acetone carboxylase	Acetone + CO_2_ + ATP + 2H_2_O → acetoacetate + AMP + 2Pi^[^ ^e^ ^]^		−39.2 ± 8.2		No^f^	Car [9]
8	Urea carboxylase	Urea + CO_2_ + ATP → urea‐1‐carboxylate + ADP + Pi		31.4 ± 11.0		No^f^	
9	Glycine cleavage system	5,10‐methylenetetrahydrofolate + CO_2_ + NH_3_ + NADH + H^+^ ⇄ glycine + H_4_folate + NAD^+^	3.2 [22], −5.6 [24]			Yes [25]	Decar [25]
10	Phosphogluconate dehydrogenase	D‐ribulose 5‐phosphate + CO_2_ + NADPH ⇄ 6‐phospho‐D‐gluconate + NADP^+^	−1.5	−10.3 ± 6.2		Yes [26]	Decar [9]
11	Isocitrate dehydrogenase (IDH)	2‐oxoglutarate + CO_2_ + NADH ⇄ isocitrate + NAD^+^	+21 [23], −5.4 [22], +11 [24]	−5.6 ± 6.2		Yes [27]	Decar [9]
12	Crotonyl‐CoA carboxylase/reductase (Ccr)	Crotonyl‐CoA + CO_2_ + NADPH ⇄ ethylmalonyl‐CoA + NADP^+^	−14.2 [22], −25.8 [24]	−39.9 ± 7.8		Yes [9]	Car [9]
13	L‐malate: NADP^+^ oxidoreductase	Pyruvate + CO_2_ + NADPH ⇄ malate + NADP^+^	−7 [28]	−12.2 ± 6.1		Yes [28]	Decar [28]
14	Formate dehydrogenase (FDH non‐metal)	NADH + CO_2_ ⇄ NAD^+^ + HCO_2_^−^	+18 [23], +13 [24]	14.2 ± 6.4		Yes [29]	Ox [29]
15	Formate dehydrogenase (FDH metal Mo‐W)	CO_2_ + 2e^−^ + H^+^⇄ HCO_2_^−^			∼ −0.40 [16, 30]	Yes [31]	Ox [32]
16	Carbon monoxide dehydrogenase (CODH)	CO_2_ + 2H^+^ + 2e^−^ ⇄ CO + H_2_O			−0.520 [33]	Yes [20]	Ox [18]
17	2‐ketoglutarate:ferredoxin oxidoreductase (2KFOR)	Succinyl‐CoA + CO_2_ + 2e^−^ ⇄ 2‐oxoglutarate + SCoA			−0.500 [34] −0.474^d^	Yes [35]	Decar [9]
18	Pyruvate:ferredoxin oxidoreductase (PFOR)	Acetyl‐CoA + CO_2_ + 2e^−^ ⇄ pyruvate + SCoA			−0.515 [36]^d^	Yes [37]	Decar [9]

^a^ ∆_r_ *G*`° calculated using equilibrator at pH 7 and 0.25 M ionic strength [38].

^b^ Reaction reported in either direction.

^c^ Thermodynamically preferred direction: carboxylation (Car), decarboxylation (Decar), or oxidation (Ox) for the reductases.

^d^ Calculated redox potential at pH 7 using equilibrator [38].

^e^ Typically 2 phosphates are released from the hydrolysis of 1 or 2 ATP molecules [39, 40], although with some enzymes, up to 4 phosphates are released [41].

^f^ Decarboxylation direction not reported.

All equations are written using CO_2_ as direct electrophile despite bicarbonate being the in‐solution substrate for some carboxylases (Table 1).

## 2 How Does Nature Utilize CO_2_?

**FIGURE 2:**
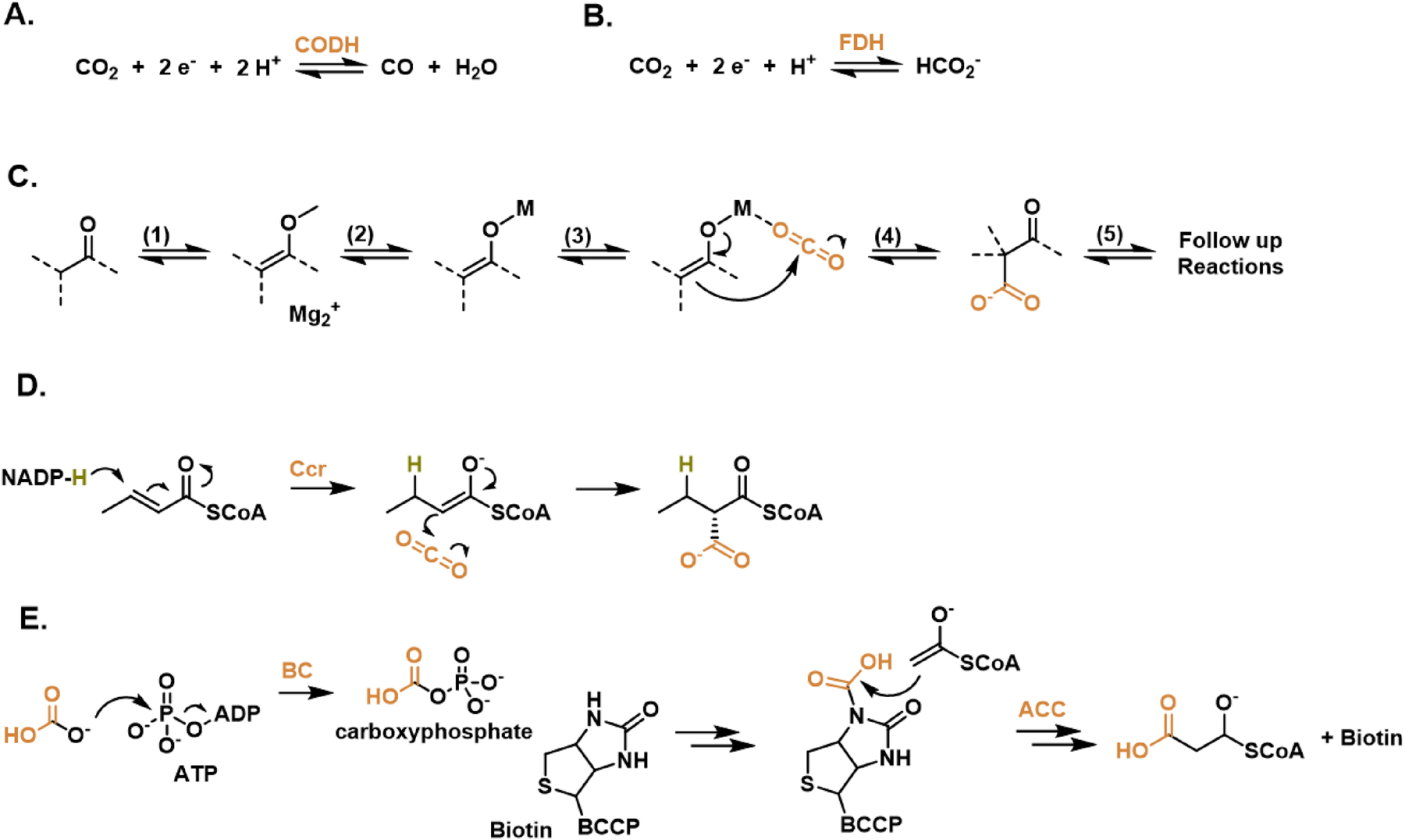
Bioelectrocatalytic CO_2_ reduction catalyzed by (A) CODH and (B) FDH. (C) Representative mechanism of biocatalytic carboxylations with (1) generation of the nucleophile, (2) stabilization of the nucleophile, (3) accommodation and/or activation of CO_2_, (4) nucleophilic attack on CO_2_, and (5) potential follow up reactions. (D) Simplified mechanism of the NADPH‐dependent carboxylation of crotonyl‐CoA by crotonyl‐CoA carboxylase/reductase [48]. (E) Simplified mechanism of the ATP‐biotin‐dependent carboxylation of acetyl‐CoA by ACC. A more detailed summary of the different carboxylation and CO_2_ reduction mechanisms can be found in [22].

We first examine the general mechanisms and thermodynamic constraints of carboxylases and reductases. Carbon dioxide‐converting enzymes operate in aqueous environments under atmospheric pressure at moderate temperatures. Biocatalytic CO_2_ transformation occurs primarily through one of two mechanisms:

- **Direct reduction**, where CO_2_ is converted by reductases such as formate dehydrogenase (FDH) and carbon monoxide dehydrogenase (CODH) (Entries 14–16 in Table 1) [22, 42, 43] into C_1_‐products such as formate or carbon monoxide [10] (Figure 2A,B). For metal‐containing FDHs and CODHs, the mechanism typically involves binding of CO_2_ to a metal active site (e.g., Ni/Fe in CODH [44, 45]) or in its close vicinity (e.g., Mo/W in FDH [46, 47]), where the metal center polarizes and activates the CO_2_ molecule. Electrons, typically delivered by ferredoxins, and protons are transferred to the metal center, enabling stepwise reduction of CO_2_. Although most FDH and CODH contain metal‐based active sites, some metal‐free FDH rely instead on NADH as a cofactor serving both for substrate activation and as hydride donor.
- **Carboxylation**, where CO_2_ is fixed by carboxylases onto an organic metabolite (Figure 2C–E). The general mechanism of carboxylation involves several key steps (Figure 2C): (1) binding of substrate and generation of a nucleophile (e.g., enol, enolate, or enamine), (2) stabilization of the nucleophile, (3) accommodation and/or activation of CO_2_ (e.g., as carboxyphosphate), (4) nucleophilic attack on CO_2_ to form a C‐C bond, and (5) potential follow up reactions such as product release from the active site [22]. Some (de‐)carboxylases deviate from this general mechanism, including pyridoxal‐5’‐phosphate (PLP)‐dependent glycine cleavage system (Entry 9 in Table 1) and the prenylated FMN cofactor‐dependent ferulic acid decarboxylases.

Enzyme‐catalyzed carboxylations are achieved by reducing the Gibbs free energy change of the reaction (Δ_r_ *G*) either by coupling with an exergonic reaction or by shifting the equilibrium according to the principle of Le Chatelier [22]. Since CO_2_ contains carbon in its most oxidized form, any functionalization must be reductive. One of the main challenges for CO_2_ utilization, aside from its activation, is therefore the supply of reducing equivalents. Here, we divide the CO_2_‐fixing enzymes into reactions being independent of external reductants, and those more relevant to bioelectrocatalysis, which require external reducing equivalents.

### 2.1 Enzymatic CO_2_ Reduction and Carboxylations Independent of External Reductants

Enzymes such as RuBisCO and PEPC (Table 1, Entries 1 and 2) are carboxylases that function without the need for external reductants. RuBisCO drives its reaction forward by coupling the carboxylation step with a hydrolysis step, leading to the formation of two carboxylic acids as low‐energy products, making the reaction energetically favorable (∆_r_ *G*° = −32.0 kJ/mol [22]). Similarly, PEPC catalyzes CO_2_ incorporation through a reaction that is energetically driven by substrate dephosphorylation, resulting in a comparable ∆_r_ *G*° of −32.2 kJ/mol [22]. In addition, reversing the reactions of decarboxylases that are independent of external reductants has enabled CO_2_ incorporation into a broad range of substrates [22]. In these cases, the reaction equilibrium was shifted toward carboxylation either by providing the carboxylating substrate in excess or by continuously removing the carboxylated product.

### 2.2 Enzymatic CO_2_ Reduction and Carboxylations Dependent on External Reductants

Other carboxylases and CO_2_‐reducing enzymes require external carriers of electrons (e.g., ferredoxins and NAD(P)H) and/or energy (e.g., ATP). Ferredoxins are redox‐active proteins containing one or more FeS clusters that act as low‐potential electron carriers, with potentials as negative as −645 mV versus SHE [49, 50]. This makes them well‐suited for driving CO_2_ reduction by FDH or CODH (Table 1, Entries 15 and 16) into formate or CO, which require potentials of approximately −420 [42, 51] and −520 mV versus SHE [43, 49] at pH 7, respectively. Ferredoxins are also natural electron donors for members of the 2‐oxoacid:ferredoxin oxidoreductase (OFOR) superfamily (Table 1, Entries 17 and 18) requiring potentials more negative than −515 mV versus SHE [49] for CO_2_ fixation.

Nicotinamide adenine dinucleotide cofactors (NAD(P)H) are hydride carriers (transferring two electrons and one proton) with a standard reduction potential of −320 mV versus SHE at pH 7 [52]. While several carboxylases utilize NAD(P)H as cofactor (e.g., Table 1, Entries 9–13), reductases are typically ferredoxin dependent except one NAD^+^‐dependent FDH (Table 1, Entry 14). However, this FDH predominantly operates in the oxidative direction due to the thermodynamic mismatch between the NADP^+^/NADPH redox couple (−320 mV vs. SHE) and the CO_2_/formate couple (−420 mV vs. SHE), leading to low CO_2_ reduction rates.

Adenosine triphosphate (ATP) is an energy carrier. Its hydrolysis drives thermodynamically unfavorable transformations by releasing between 26 and 60 kJ/mol per phosphate group hydrolyzed [22]. ATP‐ and biotin‐dependent carboxylases (Table 1, Entries 3–8) use ATP to activate HCO_3_^−^ into carboxyphosphate for the subsequent substrate carboxylation [22].

Nature employs additional complex mechanisms to overcome the thermodynamic barriers of CO_2_ utilization, often by coupling unfavorable reactions with exergonic processes other than ATP hydrolysis (e.g., hydrolysis of CoA esters, modulation of reactant concentrations). A more detailed analysis of these carboxylase mechanisms has been provided by Bar‐Even et al. [23, 52].

## 3 Specific Challenges of Bioelectrocatalytic CO_2_ Fixation

CO_2_‐fixing enzymes that derive the energy and electrons required for their reaction from the substrate operate independently of external reductants and energy sources. As a result, they are generally not suitable for direct integration into bioelectrocatalytic platforms. Indirect integration could, in principle, be achieved by coupling the carboxylation reactions to bioelectrocatalytic cascades that generate the required substrates. For example, phosphoenolpyruvate, the substrate of PEPC, can be synthesized using ATP supplied by electrochemically driven cofactor regeneration systems [53] (see Section 3.2.2). However, despite this conceptual feasibility, no bioelectrocatalytic cascade integrating PEPC‐ or RuBisCO‐based carboxylation reactions has yet been reported.

In contrast, CO_2_‐fixing enzymes that rely on external reductants or energy carriers provide a more direct interface with electrochemical systems [52]. For these enzymes, electrons can be supplied directly from electrodes by replacing the native electron donor through either direct electron transfer (DET) or mediated electron transfer (MET), or indirectly by electrochemical regeneration of the required redox cofactors (Figure 1).

### 3.1 Enzyme/Electrode Connection: DET Versus MET

Bioelectrocatalytic platforms aim for efficient electron transfer between electrodes and enzymes. In DET configurations, electrons are transferred directly from the electrode to the enzyme [54, 55] via electron tunneling. This process is highly dependent on enzyme orientation on the electrode surface, as effective tunneling requires a short distance between the electrode and the redox site of the enzyme [56, 57]. As a result, DET is typically restricted to a limited set of enzymes and electrode materials that enable favorable interfacial alignment [55]. In contrast, the MET configuration relies on redox‐active mediators (e.g., viologens, cobaltocene derivatives) that shuttle electrons between the electrode and the enzymes. When used in solution, mediators transport charges by diffusion, whereas immobilized mediators transport charges through electron hopping [58]. The choice of mediator must balance suitable redox potential, fast electron transfer kinetics, chemical stability, and compatibility with the biocatalyst to ensure energy‐efficient electron transfer. Polymers covalently functionalized with the electron mediator [59] are widely used in MET systems. These redox‐active materials enable the electrical wiring of high enzyme loading, energy‐efficient electron transfer (reversibly [60]), and simplified downstream processing (facilitated product purification) by retaining both catalysts and mediators on the electrode. Compared with DET, MET offers substantially greater flexibility in electrode material selection as electron transfer is no longer constrained by specific protein–electrode interactions or precise enzyme orientation. Both DET and MET strategies, including those based on redox‐active polymers, have successfully replaced natural electron carriers such as ferredoxin as the electron donor for several CO_2_‐reducing enzymes, including metal‐containing FDH [61, 62] and CODH [20, 63].

### 3.2 Bioelectrochemical Regeneration of NAD(P)H and ATP

The most established approach for incorporating NAD(P)H‐ and ATP‐dependent carboxylases into bioelectrocatalytic systems integrates additional enzymes such as ferredoxin‐NADP^+^ reductase (FNR) [64], diaphorases [65, 66], and aldehyde:ferredoxin oxidoreductase (AOR)‐dependent cascades [67] for the regeneration of NAD(P)H and/or ATP.

#### 3.2.1 NAD(P)H Regeneration

Due to the high cost of NAD(P)H, efficient regeneration methods are essential to achieve high total turnover numbers (TTN, defined as moles of product formed per mole of cofactor during the course of a complete reaction) for practical CO_2_ fixation with NAD(P)H‐dependent carboxylases [68]. The modification of electrodes with enzymes such as ferredoxin‐NADP^+^ reductase (FNR) [64], diaphorases [65, 69], or enzyme modules containing NAD^+^ reductase subunits [70, 71, 72] has been extensively applied for electrodriven NAD(P)H regeneration, enabling highly selective hydride transfer. Armstrong et al. immobilized FNR on mesoporous ITO electrodes and achieved efficient DET for the regeneration of NADPH, which could be combined with multiple NADPH‐dependent enzymes, reaching high TTNs at almost quantitative faradaic efficiencies [73]. The coupling of this bioelectrocatalytic NADPH regeneration with L‐malate NADP^+^ oxidoreductase (Table 1, Entry 13) enabled the fixation of CO_2_ into pyruvate to form malate [28], further used for the synthesis of aspartic acid in a 3‐step reaction cascade [74]. Similarly, FNR immobilized in a viologen‐based redox hydrogel for MET led to efficient bioelectrocatalytic NADPH regeneration coupled to crotonyl‐CoA carboxylase/reductase, showing TOFs (defined as moles of product per moles of enzyme per unit of time) for NADPH production three‐times higher than those reported using DET (employing a different FNR) [75]. The unphosphorylated cofactor NADH may be regenerated using the same approaches. For instance, Megarity et al. engineered and applied an FNR variant in DET on ITO electrodes [76], and Minteer et al. have exploited MET through the immobilization of a diaphorase in cobaltocene‐based hydrogels to drive the regeneration of NADH [65, 66].

Completely replacing the costly and fragile NAD(P)H cofactors with electrodes or artificial electron mediators would be ideal for practical electroenzymatic CO_2_ fixation. However, neither MET nor DET has yet been reported for NAD(P)H‐dependent carboxylases, and MET in NADH‐dependent FDH remains to be validated. Unlike ferredoxin, NAD(P)H functions as a hydride carrier and often as a cofactor directly involved in CO_2_ activation at the enzyme's active site. This dual role makes it particularly difficult to replace NAD(P)H with artificial electron mediators. For example, the NADH‐dependent FDH from *Candida boidinii* (Table 1, Entry 14) catalyzes the interconversion of formic acid and CO_2_. The absence of redox‐active prosthetic groups in this enzyme makes it rely entirely on NADH for both electron transfer and CO_2_ activation in vivo [77]. To bypass this dependency, one‐electron reduced viologen derivatives, either in solution [78, 79, 80, 81] or immobilized in redox polymers [82], have been explored as NADH substitutes in vitro. Yet, the catalytic rates and current densities reported for this NADH‐dependent FDH combined with viologens are significantly lower than those employing ferredoxin‐dependent (metal‐containing) FDH with artificial mediators [83]. Docking simulations have been used to search for possible mechanisms by which viologen derivatives might substitute for NADH [81, 83, 84, 85], but no explanation has been found for how viologens could enable CO_2_ activation. This leaves the feasibility of employing MET for CO_2_ reduction by NADH‐dependent FDHs largely unresolved.

#### 3.2.2 ATP Regeneration

While ATP is not an electron donor, ATP‐dependent carboxylase reactions have been recently coupled to electrode reactions for bioelectrochemical ATP regeneration to provide the needed energy or to serve as an electron sink for sacrificial reducing agents. This approach is advantageous in atom‐economy over traditional enzymatic methods, which rely on kinases and stoichiometric high‐energy sacrificial phosphate donors [86, 87]. In these systems, the electrode supplies energy, but electrons are not directly needed in ATP regeneration. Consequently, the electrochemical reaction can be either anodic or cathodic, depending on the specific regeneration mechanism.

**FIGURE 3:**
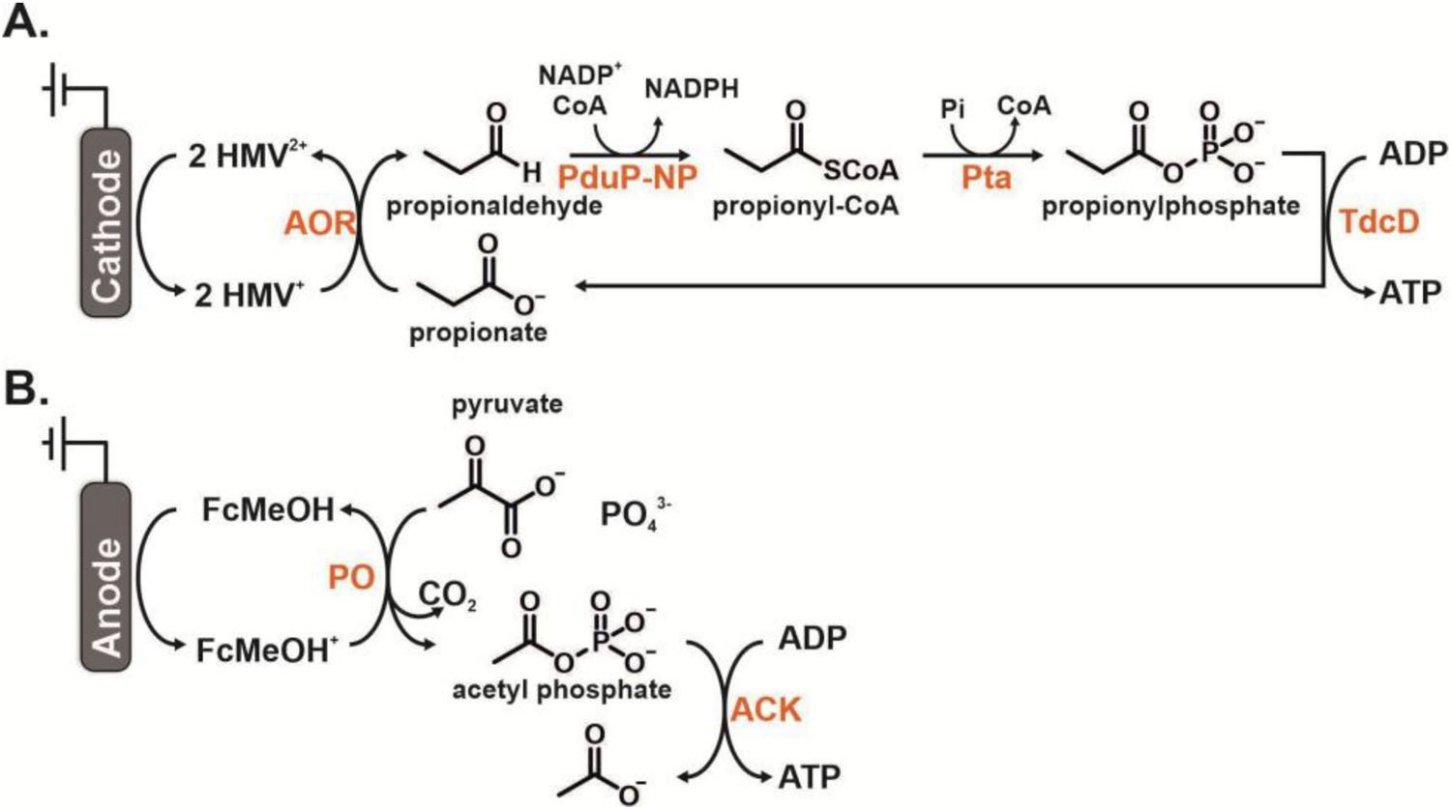
Cathodic (A) and anodic (B) bioelectrocatalytic ATP regeneration platforms, adapted from references [67, 87]. The cathodic regeneration is solely driven by the electrode reaction while the anodic cascade needs a sacrificial reducing agent (pyruvate).

Luo et al. recently reported the first cathodic bioelectrocatalytic regeneration of ATP (and NADPH) [67] via a cyclic four‐step enzymatic cascade (Figure 3A). This platform enables the regeneration of propionaldehyde from propionate (*E*° acid/aldehyde = −580 mV vs. SHE) by supplying electrons to a ferredoxin‐dependent aldehyde oxidoreductase (AOR_Aa_) using hexamethyl viologen (*E*° HMV^2+^/HMV^+^ = −610 mV vs. SHE) as an artificial redox mediator. The oxidation of propionaldehyde back to propionate, catalyzed by CoA‐acylating propionaldehyde dehydrogenase (PduP‐NP), phosphate acetyltransferase (Pta), and propionate kinase (TdcD) (Figure 3A), releases NADPH and ATP. Up to 0.4 mM ATP was produced from 1 mM ADP and 60 mM propionate with a rate of 1.03 µmol cm^−2^ h^−1^ for the electron‐driven ATP production, and a Faraday efficiency of 22%. The platform was applied for the synthesis of glucose 6‐phosphate (G6P) with a production rate of 2.7 µmol cm^−2^ h^−1^ after 1 h of electrolysis (400 µM G6P produced, 20% yield, 47% Faraday efficiency, from 2 mM glucose), higher than the uncoupled ATP production rate attributed to the in situ regeneration of the formed ATP by the hexokinase to form G6P. The system provides a proof of concept for ATP regeneration that, in principle, could bypass the need for sacrificial reagents and thereby achieve high atom economy. However, the very low total turnover numbers (TTN_ADP_ = 0.4) highlight the need for substantial improvement before this strategy can be considered a practical atom‐economic ATP recycling approach.

Anodic bioelectrocatalytic ATP regeneration has also been developed, leveraging the properties of pyruvate oxidase (PO), which catalyzes the phosphorylation and decarboxylation of pyruvate to acetyl phosphate, a high‐energy phosphate donor used by acetate kinase (AcK) for the synthesis of ATP (Figure 3B) [53, 87]. This approach replaces O_2_ as the natural electron sink of PO with artificial electron acceptors (e.g., ferrocene derivatives), eliminating challenges related to low concentration of dissolved O_2_ and the need for catalase to remove H_2_O_2_ generated from O_2_ reduction. Siedentop et al. reported the anodic production of 4.12 mM ATP from 15 mM ADP and 200 mM pyruvate after 18 h, corresponding to a rate of 0.3 µmol cm^−2^ h^−1^ [53]. In comparison, when the electrochemical system is turned off, only half the amount of ATP is produced using O_2_ as an electron acceptor. They demonstrated the application of the platform for the phosphorylation of mevalonate with up to 6.8 mM mevalonate phosphate produced within 18 h (using 15 mM mevalonate, 0.1 mM ADP, and 200 mM pyruvate), corresponding to a rate of 0.5 µmol cm^−2^ h^−1^ and a TTN_ADP_ of 68. Additionally, they extended the cascade by incorporating a polyphosphate kinase (PPKs), specifically AjPPK2, which converts AMP into ADP, thereby broadening the substrate scope of the platform. On a similar approach, Ruccolo et al. [87] reported the phosphorylation of 2‐ethynylglycerol (TTN_ADP_ 82) and creatine (TTN_ADP_ 28), the ligation of serine and phenylalanine (TTN_ADP_ 35), and the synthesis of guanosine‐5`‐triphosphate (TTN_ADP_ 87) [87]. Scalability of the platform was showcased in a flow reactor (256 cm^2^ working electrode) for the synthesis of molnupavir in an ATP‐dependent two‐enzyme reaction, processing > 20 g substrate and achieving a rate of 13.7 µmol cm^−2^ h^−1^ (productivity of 1.1 g L^−1^ h^−1^) at a current density of 1.5 mA cm^−2^ with a faradaic efficiency of 76% and a TTN_ADP_ of 94. These metrics are based on analytical yields; the product has not been isolated and purified.

An alternative anodic electroenzymatic ATP regeneration strategy was reported by De Lacey and coworkers [88, 89], utilizing an electrochemically induced proton gradient to drive the cofactor regeneration at an ATP synthase. In this system, a phospholipid bilayer containing a [NiFeSe]‐hydrogenase and F_1_F_0_‐ATP synthase was deposited on a gold electrode. The bilayer serves as an enzyme support, creating a confined aqueous phase near the electrode where bioelectrocatalytic H_2_ oxidation induces a local pH shift of 1–2 units, generating the necessary proton gradient for ATP synthesis. Over 30 µM ATP was produced in the presence of 0.5 mM ADP and 1 atm H_2_ with a calculated rate of 4.5 µmol cm^−2^ h^−1^ (TOF = 1800 s^−1^) for the gradient driven platform [89]. The platform's application was demonstrated with the phosphorylation of glucose into G6P and NAD^+^ into NADP^+^ with approximate calculated rates of 6.7 µmol cm^−2^ h^−1^ (> 0.1 mM G6P formed, 5.3 µmol G6P s^−1^ mg^−1^ ATPase, from 0.5 mM ADP and 20 mM glucose) and 0.2 µmol cm^−2^ h^−1^ (> 10 µM NADP^+^ formed, 0.08 µmol NADP s^−1^ mg^−1^ ATPase, from 1 mM ADP and 10 mM NAD^+^).

In assessing the economic feasibility of ATP recycling, Siedentop et al. [53]. defined benchmark values of TTN_E_ > 10^4–^10^5^, TOF > 1 s^−1^, TTN_cofactor_ > 10^2–^10^5^ and space‐time yields (STY) > 2 g L^−1^ h^−1^. The pyruvate‐based approach comes close to meeting some of these thresholds, but its viability is constrained by the reliance on pyruvate as a sacrificial reducing agent and the release of acetate as byproducts, which reduces the atom economy of the regeneration platform. More critically, the decarboxylation of pyruvate releases one CO_2_ molecule for each CO_2_ fixed, resulting in no net CO_2_ fixation, or even releases more CO_2_ than is fixed if the carboxylase reactions require more than one ATP or the full conversion of ATP to AMP (Table 2, Entry 7). By contrast, the cathodic ATP regeneration methods, which avoid sacrificial agents, and the gradient‐driven platform, which consumes only H_2_ as a sacrificial reducing agent, represent potentially more suitable alternatives for carboxylation reactions. However, both methods currently produce only substoichiometric amounts of product (TTN_ADP_ < 1), hindering their applicability. A further barrier to deployment is the complexity of the multienzyme cascades required for ATP regeneration, in stark contrast to the comparatively simpler solutions available for NAD(P)H regeneration. To date, no ATP‐dependent carboxylase (Table 1, Entries 3–8) has yet been coupled to any of these electrochemical ATP recycling platforms.

### 3.3 Catalytic Rates—Current Densities

High catalytic currents are a primary objective in bioelectrosynthetic platforms, as they directly correlate with high production rates (mol product per cathode area per time) [90] and STY (mol product per volume per time) [91]. The catalytic currents are influenced by the intrinsic activity and loading of the enzyme [92, 93], by substrate transport, and by electron transfer rates. The electron transfer rate depends on the specific mode of electron communication, either DET via direct electrode‐enzyme interactions or MET through enzyme‐redox mediator interactions. While enzymes are typically immobilized on the electrode, configurations with freely diffusing enzymes in solution are also possible.

Here we examine the limitations and challenges in achieving high current densities in bioelectrocatalytic CO_2_ utilization platforms, including the intrinsic kinetic parameters of the enzymes, potential mass transfer limitations arising from the low solubility of CO_2_ in aqueous buffers, and the impact of controlled reaction environment, such as confinement effects that can lead to changes in the local concentration of substrates, protons, intermediates, and products.

#### 3.3.1 CO_2_‐Fixing Enzymes Reaction Kinetics

Bar‐Even et al. previously conducted a comprehensive literature survey on the kinetic properties of some carboxylases [10]. They highlight the need for high *k*_cat_ and *k*_cat_/*K*_M_ values, which are critical for achieving high reaction rates under either saturating or low CO_2_ concentrations. The most efficient carboxylases, such as PEP carboxylase (Table 1, Entry 2, *k*_cat_ 150 s^−1^, *k*_cat_/*K*_M_^CO2^ 1.7 × 10^7^ s^−1^ M^−1^), pyruvate carboxylase (Table 1, Entry 5, *k*_cat_ 90 s^−1^, *k*_cat_/*K*_M_^CO2^ 7.7 × 10^5^ s^−1^ M^−1^) and crotonyl‐CoA carboxylase/reductase (Table 1, Entry 12, *k*_cat_ 80 s^−1^, *k*_cat_/*K*_M_^CO2^ 4.1 × 10^5^ s^−1^ M^−1^) exhibit catalytic performances well above those of the “average enzyme” (*k*_cat_ ∼10 s^−1^ and *k*_cat_/*K*_M_ ∼10^5^ s^−1^ M^−1^) [94]. Some of the best CO_2_‐reducing enzymes, such as CODHs (Table 1, Entry 16, *k*_cat_ 420 s^−1^) and metal‐dependent FDHs (Table 1, Entry 15, *k*_cat_ 46.6 s^−1^, *k*_cat_/*K*_M_ ～3 × 10^6^ s^−1^ M^−1^ with MV as electron donor [95]), display kinetic properties comparable to the fastest carboxylating enzyme classes [10] (Table 1, Entries 15, 16). These catalytic efficiencies may even be significantly underestimated, at least in the case of CODH, because protein film electrochemistry experiments showed that maximal TOFs for the reduction of CO_2_ are actually similar to those observed for the oxidation of CO (bias CO oxidation/CO_2_ reduction = 0.6), although the solution assays suggest that CO_2_ reduction is much slower (bias > 71) [17].

The high *k*_cat_ values and ability to operate without cofactors make the metal‐dependent FDHs and CODH particularly effective in bioelectrocatalytic CO_2_ reduction, whether under DET or MET. Edwardes et al. [96] reported current densities up to −3.6 mA cm^−2^ at −0.6 V versus SHE for CO_2_ reduction to formate with a W‐FDH under DET in an inverse‐opal titanium dioxide electrode with 96 % faradaic efficiency and a TOF of 28 s^−1^. Under MET, Kano et al. achieved current densities up to 20 mA cm^−2^ using W‐FDH immobilized on a Ketjen black modified electrode with triquat as diffusible electron mediator [61]. Sahin et al. reported the application of formylmethanofuran dehydrogenase for the reduction of CO_2_ to formate under DET with current densities up to 120 µA cm^−2^ and quantitative Faradaic efficiency [97]. Engineered CODH from *R. rubrum* immobilized on gas diffusion electrodes under DET has achieved current densities of up to 4.2 mA cm^−2^ for CO_2_ reduction at −0.8 V versus SHE with a TOF of 420 s^−1^. Under MET, the immobilization of CODH from *Carboxydothermus hydrogenoformans* in a cobaltocene‐based redox polymer enabled current densities up to 5.5 mA cm^−2^ on gas diffusion electrodes with a TOF of 2.7 s^−1^ [63].

For cofactor‐dependent carboxylases, we have demonstrated the bioelectrocatalytic application of Ccr (Table 1, Entry 12) co‐immobilized with FNR in a viologen‐based redox polymer [75]. This MET system enables electron‐driven CO_2_ fixation into crotonyl‐CoA, achieving currents up to 150 µA cm^−2^ and a Ccr‐specific activity of 1.4 U mg^−1^ (TOF of 1.2 ± 0.3 s^−1^) starting from 1 mM crotonyl‐CoA with 200 µM of NADP^+^. However, this activity represents just over 1% of the activity obtained for the enzyme in solution (110 U mg^−1^[98]), indicating that the overall catalytic rate is limited by cofactor regeneration. Armstrong and coworkers [74] further demonstrated the coupling of FNR in DET and the NADPH‐dependent malic enzyme “ME” (Table 1, Entry 13) within an enzymatic cascade including fumarase and aspartate‐amino‐lyase connecting pyruvic acid with L‐aspartic acid. This system achieved a current density of up to 25 µA cm^−2^ and a 34% conversion over 20 h electrolysis (1.5 µmol h^−1^) using 20 µM NADP^+^ (TTN of 340 aspartate/NADP^+^). The co‐immobilization of only FNR/ME led to CO_2_ fixation rates of 7 s^−1^ [28], higher than the previously reported uncoupled NADP^+^ reduction rate of 0.4 s^−1^ [66].

The achievable current densities for cofactor‐dependent carboxylases are often limited by the intrinsic activity of the cofactor recycling platform. While the cofactor regenerating enzymes are fast, their integration on electrodes often leads to low TOF. For instance, FNR for NADPH production in solution has a *k*_cat_ of 200–600 s^−1^[99, 100] (and *K*_M_^NADP^ in the range 2–40 µM [100]) compared to the observed bioelectrocatalytic TOFs of 0.4 s^−1^ in DET [64] and 1.2 s^−1^ in MET [75]. The lower kinetic properties of immobilized redox enzymes (either cofactor regenerating or CO_2_ reductases) compared to the activities from solution assay, especially for fast cofactor regenerating enzymes such as FNR, are typically associated with limitations by mass transfer [101], by electron transfer between the enzyme and the redox partner [17], and/or conformational changes of the immobilized enzyme, including denaturation [102]. Differences may also be explained by overestimation of how much the enzyme contributes to the current, which could lead to underestimation of the apparent kinetic properties of the immobilized enzyme. Nevertheless, higher reaction rates are typically achieved with cofactor regeneration platforms when coupled to cofactor‐dependent carboxylases in comparison to the rates observed for the uncoupled cofactor production. The effect is attributed to the product removal through downstream reactions, enhancement through confinement effect (see Section 3.3.3), or prevention of the accumulation of unstable redox forms of the cofactors (e.g., NAD(P)^+^ is more stable than NAD(P)H [103]).

#### 3.3.2 Nature of the Substrate of CO_2_ Fixing Enzymes

Compared to conventional electrocatalytic CO_2_ reduction platforms, where low CO_2_ partial pressures lead to reduced current densities and selectivities [3], bioelectrocatalytic CO_2_ fixation benefits from the low *K*_M_ values of enzymes (Table 1). This enables rapid and selective CO_2_ fixation even at low gas concentrations (the Henry's constant of CO_2_ in water is 34 mM atm^−1^) [7, 104].

A fundamental aspect of optimizing bioelectrocatalysis and minimizing mass transport limitations is determining whether the substrate of the enzyme is CO_2(aq)_ or bicarbonate. In aqueous solutions, CO_2_ exists in equilibrium between its solvated and hydrated forms. Since the apparent pKa for the CO_2_/HCO_3_^−^ couple is 6.4, both CO_2_ and bicarbonate are present in significant amounts at physiological pHs. This equilibrium makes it impossible to discriminate between the substrates based on steady‐state experiments.

Experimental methods for identifying the substrate exploit the fact that CO_2_/HCO_3_^−^ interconversion is slow (about 20 s at 25°C and pH 7). By preparing solutions containing either pure CO_2_ (in acidic conditions) or bicarbonate (in basic conditions) and monitoring enzymatic activity immediately after injection of one of the pure forms of the substrate, before the other form of CO_2_ accumulates, it is possible to determine which is the substrate. Early studies leveraged the significantly slower equilibration at low temperatures, typically employing isotopic labeling techniques in combination with UV‐visible spectroscopy and NMR analysis [11, 105, 106].

**FIGURE 4:**
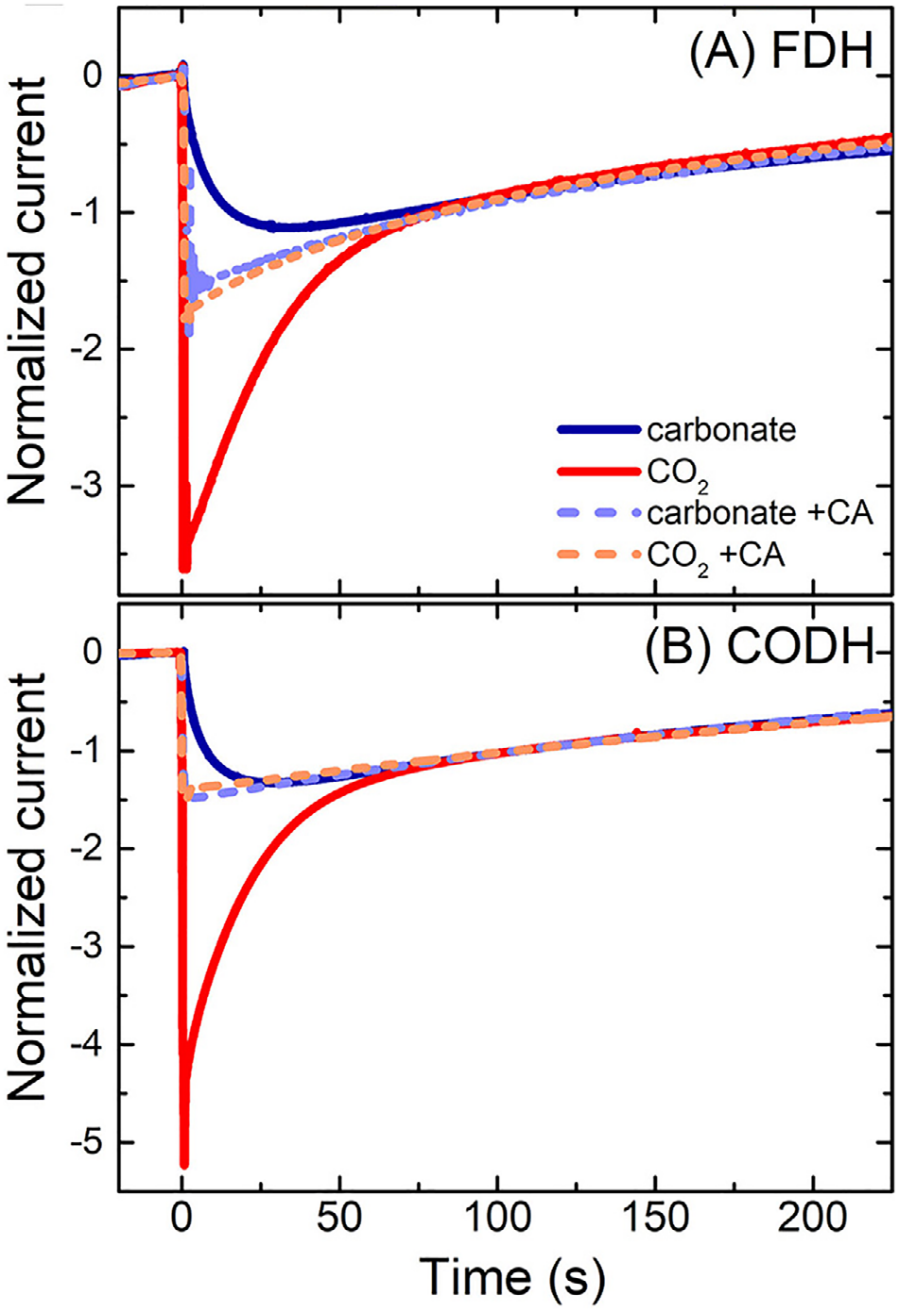
(A) reductive catalytic current of a film of *Desulfovibrio vulgaris* formate dehydrogenase immobilized on a graphite electrode upon addition of carbonate and CO_2_ in the absence of carbonic anhydrase (blue and red, respectively) and in the presence of carbonic anhydrase (dotted light blue and dotted light red, respectively). Immediate appearance of the reduction current is only observed upon addition of CO_2_ or in the presence of carbonic anhydrase, demonstrating that CO_2_ is the substrate. (B) same experiments as in panel A, but conducted on a film of *Thermococcus sp. AM4* CODH 2, with the same color code and similar conclusions. Reproduced from [107] with permission.

Recently, we adapted this approach to protein film electrochemistry experiments and demonstrated that CO_2_, not HCO_3_^−^, is the substrate for the two enzymes that catalyze the direct reduction of CO_2_: metal‐bound FDH and CODH [107] (Figure 4). This method is directly applicable to any redox enzyme interacting with CO_2_ under DET conditions, and can be adapted to MET. Moreover, it holds potential for resolving the true substrate of various carboxylases where this is not yet validated experimentally (e.g., acetone carboxylase [39, 108], Table 1, Entry 7), provided that a catalytic current can be detected.

#### 3.3.3 Bioelectrocatalysis under Confinement

**FIGURE 5:**
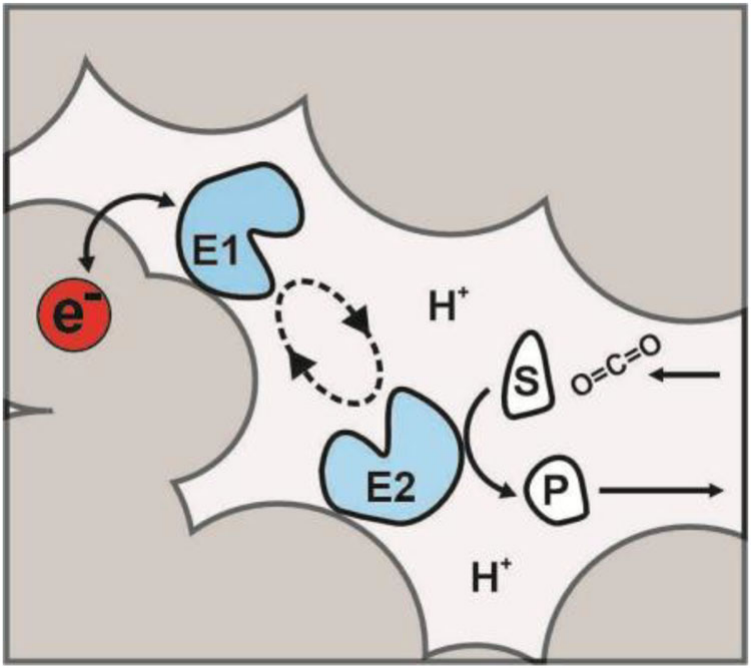
Bioelectrocatalytic cascades in confined microenvironments.

In all living cells, enzymes operate within the confinement of cellular compartments, which serve to locally concentrate substrates and enzymes, retain cofactors and reaction intermediates, and create optimized microenvironments in terms of pH, redox state, or cofactor pools [109]. Cells are highly crowded, with macromolecule concentrations reaching up to 400 g L^−1^, occupying between 5% and 40% of the total volume [110]. Crowding influences not only protein association and stability but can also modulate enzyme kinetics (*k*_cat_ and *K*_M_) due to crowding‐induced conformational changes [73, 111, 112]. From both a fundamental and technological perspective, engineering artificial confined enzymatic cascades is therefore of great interest [113]. Confinement is particularly advantageous in bioelectrocatalytic platforms for NADPH regeneration coupled to cofactor‐dependent carboxylases, where it helps address the trade‐off between achieving high current density and minimizing NAD(P)H costs by operating at low cofactor concentrations. Indeed, electrochemically driven enzyme cascades confined within microstructured electrodes (Figure 5) or embedded in redox‐active films have already been shown to significantly enhance reaction rates.

Armstrong et al. [73]. developed a bioelectrocatalytic platform for NADPH regeneration coupled to NADPH‐dependent cascades within porous ITO electrodes. The confinement of NADPH/NADP^+^ within the porous electrode led to fast recycling of the cofactor, reducing the required cofactor concentration in the bulk solution down from 0.1–5 mM to just 5–20 µM [73]. In the case of the oxidation of isocitrate to 2‐oxoglutarate using co‐immobilized FNR and IDH, the system could even be operated using the very low amount of NADPH (153 pmol) carried as cargo by the IDH itself without added cofactor in the bulk solution, leading to an outstanding total turnover number (TTN > 150000) for cofactor regeneration [114]. Further applications of this confinement approach include deracemization, NADPH‐ and ATP‐dependent cascades, and the scale up of NADPH‐dependent enzymatic transformations [73].

We have also exploited the confinement effect by co‐immobilizing FNR and Ccr within a viologen‐modified polymer film on glassy carbon electrodes for the carboxylation of crotonyl‐CoA. The high local concentration of the enzymes within the redox‐active polymer matrix resulted in significantly higher reaction rates compared to the use of Ccr as a freely diffusing enzyme in the electrolyte. The enhanced cofactor cycling due to confinement enabled the CO_2_‐fixing cascade to operate with 20 µM NADP^+^, achieving current densities of 140 µA cm^−2^. The system could even be used with as little as 2 µM cofactor, although current densities decreased to 20 µA cm^−2^ [75].

Cobb et al. [115]. exploited enzyme confinement to mimic the carbon‐concentrating mechanism of carboxysomes, co‐immobilizing FDH and carbonic anhydrase (CA) within mesoporous ITO electrodes to achieve CO_2_ reduction at atmospheric concentrations, converting it into formate. CA maintained a constant concentration of CO_2_ or bicarbonate through rapid interconversion, with TOFs reaching 10^6^ s^−1^. Using finite element modeling, they optimized buffer capacity and pH to increase local HCO_3_^−^ concentrations, which CA then converted into CO_2_ within the porous electrode. This approach mitigated diffusion limitations and substrate depletion, enabling efficient utilization of the total dissolved carbon pool. The beneficial role of CA in enhancing CO_2_ reduction yields has also been observed in systems using multienzymatic cascades for methanol synthesis from CO_2_ [22].

Although confining high enzyme loadings on electrodes is beneficial for achieving high current densities, the interfacial nature of bioelectrocatalysis can also give rise to pronounced local gradients in substrate and product concentrations. At high turnover rates, these gradients may decouple local conditions from those of the bulk electrolyte. For example, during bioelectrocatalytic CO_2_ reduction (Table 2, Entries 15 and 16) or regeneration of the NADPH required by NADPH‐dependent carboxylases (Table 2, Entries 9–14), protons are consumed near the electrode surface. If this consumption outpaces proton replenishment from the bulk solution, localized pH increases can occur, potentially impairing enzyme activity. Edwardes et al. [96]. demonstrated the change in local pH under turnover using immobilized FDH in mesoITO and hierarchical IO‐TiO_2_ electrodes. The CO_2_ reduction to formate, which involves a net proton consumption (Table 2, Entry 15), resulted in an increase of up to 2 pH units due to the enzymatic catalysis under confined conditions. Finite element modeling incorporating enzymatic kinetic parameters, current densities, and electrode architecture, successfully guided the selection of conditions (buffer pKa and bulk pH) to achieve optimum local pH for maximal activity of the immobilized enzyme.

In a separate study, Siritanaratkul applied a one‐dimensional reaction–diffusion model to investigate porous electrodes containing immobilized enzyme cascades, including NAD(P)H‐dependent enzymes and cofactor regeneration systems [116]. The results showed that increasing the rates of the NAD(P)H‐dependent enzyme leads to higher achievable current densities until substrate transport from the bulk becomes rate‐limiting due to rapid depletion near the electrode. This highlights the critical role of cell and electrode design in alleviating mass‐transfer limitations, for example, through the use of gas diffusion electrodes (Section 3.3.1) and flow electrolyzers.

### 3.4 Energy Efficiency: Reversibility and the Role of Enzymes

In chemistry, the term *reversibility* is used with two distinct meanings [117]: in chemical kinetics, it refers to reactions or steps that can proceed in both directions, whereas in thermodynamics, it describes transformations that occur close to equilibrium and therefore without energy dissipation. In the context of catalysis, it is essential to clearly distinguish between these two concepts. Accordingly, the term *bidirectionality* is now used to describe whether a reaction proceeds in both directions, while *reversibility* is reserved for its thermodynamic meaning [57, 118]. A reaction can therefore be considered reversible only if it proceeds at appreciable rates in response to very small deviations from equilibrium, that is, with a small free energy of reaction. Understanding why certain redox catalysts are able to operate reversibly remains an active area of research in electrochemistry [119, 120, 121, 122, 123, 124]. This question also has relevance in biocatalysis and bioelectrocatalysis, where approaching thermodynamic reversibility is essential for maximizing energy conversion efficiency [118].

In the context of electroenzymatic CO_2_ fixation, we distinguished the homogeneous reactions where electrons are exchanged between different molecules (e.g., between NAD(P)H, CO_2_ and the substrate of NAD(P)H‐dependent carboxylases) and the heterogeneous reactions where electrons are transferred to or from an electrode (e.g., CO_2_ reduction at FDH or CODH in DET with electrodes). For the former, we have compiled reaction Gibbs free energies (∆_r_ *G*'°) that have been reported in the literature or calculated using Equilibrator [38] (a web interface that enables the calculation of Gibbs energies of reactions) (Table 2, Entries 1–8 and 10–14). For the latter, we indicate in the same table the reduction potentials of the reactions at pH 7 (Table 2, Entries 15–18). In both cases, homogeneous and heterogeneous reactions, a very negative ∆_r_ *G* makes the reaction irreversible. This large free energy is used to drive the reaction forward, and it is dissipated as heat.

For homogeneous reactions, the reaction free energy is determined by the standard value but also depends on the concentrations of substrate and product. A tenfold change in the reactant‐to‐product concentration ratio corresponds to a change of 6 kJ/mol in free energy. Consequently, reactions with small ∆_r_ *G* (e.g., ATP‐independent carboxylases, Table 2, Entries 10, 11, and 13) can have either negative or positive reaction free energies depending on the concentrations of reactants. These reactions may therefore proceed in either direction, on condition that the catalyst allows bidirectionality. Considering that most of those enzymes act as decarboxylases under biological conditions (Table 2), their bidirectionality would allow for their utilization in artificial CO_2_ fixation platforms.

For heterogeneous reactions, the reaction free energy depends on the difference between the electrode potential (which can be changed at will, and over a large range) and the Nernst potential of the redox couple (calculated from the standard value and the concentrations of oxidized and reduced reactants using the Nernst equation). As examples of the heterogeneous case, certain metal‐dependent FDHs [125] and all CODHs [57, 125] characterized so far (Table 2, Entries 15 and 16) behave bidirectionally and reversibly when connected to electrodes under conditions of DET, enabling their use for energy efficient reduction of CO_2_ into formate or CO, respectively.

As examples of the homogeneous reactions coupled to a heterogeneous reactions, Armstrong and coworkers demonstrated that FNR in DET in nano‐porous electrodes (heterogeneous case) co‐immobilized with NADPH‐dependent carboxylases (homogeneous case) can be operated as catalytically reversible cascades. For instance, IDH (Table 1, Entry 11), which is typically used in the decarboxylation direction, could be driven under reversible catalysis when coupled to the electroenzymatic NADPH/NADP^+^ regeneration [27, 126]. In a second example, the reversible behavior of co‐immobilized malic enzyme (Table 1, Entry 13) within the porous electrodes modified with FNR served to demonstrate the reversibility of a multi‐enzyme cascade, also including fumarase and L‐aspartate ammonia‐lyase, for connecting pyruvic acid with L‐aspartic acid [74].

Another example of homogeneous reactions involves enzymes from the OFOR family, which can be driven electrochemically in solution via MET using ferredoxin as a freely diffusing electron mediator [35, 49, 127]. Employing ferredoxins with different redox potentials enables bidirectional control of the OFOR reaction, directing it toward either carboxylation or decarboxylation. Some OFOR–ferredoxin combinations can even catalyze both directions near the equilibrium potential [35, 49, 127], indicating reversibility of the system. The OFOR‐catalyzed carboxylation reactions are typically driven by the hydrolysis of a thioester bond in a CoA ester substrate (with the exception of oxalate oxidoreductase [128]). Consequently, although the system behaves thermodynamically reversibly, electroenzymatic CO_2_ fixation by OFORs is not solely driven by electrochemical energy.

### 3.5 O_2_ Sensitivity

While ferredoxin‐dependent CO_2_‐fixing enzymes are generally O_2_‐sensitive (Table 1, Entries 15–18), the other CO_2_‐fixing enzymes are typically O_2_ tolerant. Nevertheless, O_2_ remains a major challenge for all electroenzymatic CO_2_ fixation approaches considering that the low potentials required for either CO_2_ reduction (Table 2, Entries 15–18) or for NAD(P)H/ATP regeneration promote O_2_ reduction both at the electrode and via redox mediators such as viologens [129]. This side reaction not only decreases faradaic efficiency but also generates reactive oxygen species (ROS), which may inactivate enzymes and degrade substrates, products, and cofactors [130]. For O_2_‐sensitive CO_2_‐fixing enzymes, O_2_ exposure is even more detrimental, as it can cause irreversible deactivation even in the absence of an applied potential [131].

#### 3.5.1 Natural O_2_ Protection Mechanisms

Many CO_2_‐fixing enzymes have evolved mechanisms to withstand aerobic conditions. These include accessory domains that shield vulnerable radical or metal centers, structural modifications to iron cofactors that make them less prone to oxidation, and, in some cases, the replacement of iron with alternative metals [131]. Among CO_2_‐fixing enzymes, a subset of pyruvate‐ferredoxin oxidoreductases (PFORs, Table 1, Entry 18) features an additional C‐terminal extension containing two cysteine residues that form a disulfide bond upon O_2_ exposure. This disulfide prevents electron transfer within the protein, suppressing unintended electron transfer to O_2_. Enzyme activity can be restored in vitro by reducing the disulfide bond with thiols [131]. A similar protection mechanism was recently identified in a FDH from *Desulfovibrio vulgaris* (W/Sec‐FdhAB) which, unlike most O_2_‐sensitive metal‐dependent FDHs, can be handled aerobically in an inactive resting state [132]. In the CODH from *D. vulgaris*, the natural substitution of a typical [4Fe‐4S] cluster with an oxygen‐resistant [2Fe‐2S] cluster, enhances O_2_‐tolerance [133]. Blocking the channel that is used to guide the diffusion of CO and O_2_ in CO‐dehydrogenase can increase O_2_‐resistance, but simultaneously slows CO access to the active site [134, 135]. While these adaptations facilitate enzyme expression and handling, they do not enable turnover in the presence of O_2_, necessitating strategies to maintain strictly anoxic conditions during catalysis.

Nature has also evolved carbon‐concentrating mechanisms to locally increase CO_2_ concentrations. For instance, bacterial carboxysome encapsulates CA and RuBisCO within a protein shell that selectively allows HCO_3_^−^ to enter while preventing CO_2_ escape, potentially also limiting O_2_ entry [136, 137]. Within the carboxysomes, CA converts HCO_3_^−^ into CO_2_ increasing its local concentration and enhancing RuBisCO's carboxylase activity while suppressing its competing oxygenase activity. Liu et al. [138] leveraged this concept by encapsulating an O_2_‐sensitive FeFe‐hydrogenase in carboxysomes alongside ferredoxin and FNR to create a nanoreactor for H_2_ production. Compared to free hydrogenases, this NADPH‐ or methyl viologen‐powered nanoreactor exhibited a 20% increase in H_2_ evolution activity and improved storage stability under aerobic conditions. The authors proposed that the carboxysome shell, which selectively permits entry of negatively charged metabolites and protons, prevents the entry of O_2_, thereby protecting the hydrogenase. This strategy of compartmentalization and localized CO_2_ concentration could similarly benefit bioelectrocatalytic CO_2_ fixation.

#### 3.5.2 Artificial O_2_ Protection Mechanisms

O_2_ scavengers [139, 140, 141] and deoxygenation methods [142] are often impractical for bioelectrocatalytic CO_2_ fixation due to the requirement for (super)stoichiometric reducing equivalents or their high energy demands. As an alternative, we have proposed the use of redox‐active films to create localized anaerobic conditions, enabling O_2_‐sensitive enzymes to function in bioelectrocatalytic systems even in the presence of O_2_. We demonstrated that immobilizing an O_2_‐sensitive hydrogenase within a viologen‐modified film allows electrons from H_2_ oxidation to be transferred to the viologens, which subsequently reduce O_2_. This process depletes O_2_ within the film, effectively protecting the embedded enzyme, enabling continuous operation under aerobic conditions [143, 144, 145]. The time scale of protection scales exponentially with film thickness, providing quasi‐infinite protection for films as thin as 10 µm [146]. However, O_2_ reduction via viologens produces H_2_O_2_, which can degrade the system. To mitigate this, we used a peroxide‐dismutating catalyst, such as iodide [129], preventing oxidative damage to both the enzyme and the film. The use of redox‐active films was later extended to FDH for formate oxidation to CO_2_ in presence of O_2_ [147] demonstrating the broad applicability of protecting films for enabling O_2_‐sensitive bioelectrocatalytic transformations. However, their application in electroenzymatic CO_2_ fixation with O_2_ sensitive enzymes has yet to be explored.

## 4 Summary and Outlook

Electroenzymatic CO_2_‐fixation has emerged as a promising strategy for utilizing CO_2_ as feedstock for the sustainable synthesis of value‐added products. This process enables chemo‐, regio‐ and stereoselective CO_2_ reduction under mild conditions with high conversion rates. The vast and still largely unexplored biodiversity of CO_2_‐converting enzymes supports a wide range of applications, from producing small C_1_ commodity chemicals to enabling the selective synthesis of complex products, an advantage over conventional electrocatalytic CO_2_ reduction, which lacks selectivity for C_2+_ products. Recent advances in bioelectrocatalytic CO_2_‐fixation have achieved current densities in the mA cm^−2^ range and sustained turnover over several days. Enzyme screening and engineering efforts have prioritized high catalytic rates and robustness. Additionally, advances in enzyme confinement on electrodes have maximized loading, improved management of cofactors and intermediates, and stabilized the enzyme. These combined strategies have led to high total turnover numbers.

However, electroenzymatic CO_2_ fixation remains at a low technology readiness level. Substantial improvements in current densities and operational stability are required before these systems can compete with conventional, fossil‐based chemical processes. Scaling up also remains a major challenge. Electrode materials must be free of critical raw materials. This is straightforward for mediated electron transfer, where electrodes mainly act as conductive supports and allow for cost‐effective carbon‐based materials to be used. In contrast, direct electron transfer can be more challenging, as efficient electron transfer to the enzyme typically requires surface engineering [148] and, in some cases, interfaces based on critical raw materials (e.g., ITO for FNR [64]). Enzymes are largely composed of abundant elements, with some exceptions such as Ni, Mo, or W in certain CO_2_‐reducing enzymes (e.g., CODH and FDH). Nevertheless, enzyme production can be challenging due to low yields; for example, the FDH from *Nitratidesulfovibrio vulgaris* Hildenborough [132], which performs exceptionally well on electrodes, can currently only be produced in its native host, restricting scalable production. An even greater scalability challenge arises from the need for small‐molecule cofactors for electron and energy transfer. While some reductases (e.g., FDH, CODH and the OFOR family) accept electrons directly into the CO_2_ reduction mechanism, most carboxylases involved in producing complex value‐added chemicals depend on external cofactors, introducing a major bottleneck in cost and robustness associated with NAD(P)H and ATP. Despite recent advances, bioelectrocatalytic platforms for cofactor regeneration still require significant improvements (especially for ATP) before they can be considered practical recycling solutions.

Future technology breakthroughs may rely on enzyme engineering, including de novo enzymes, for creating CO_2_‐fixing enzymes that are independent of cofactors. Additionally, optimizing enzymes for high current density conditions and designing electrochemical systems tailored to enzyme stability and activity will be essential. Achieving optimal performance may benefit from the co‐design of enzymes, electron mediators, and electrode materials to maximize product yield while minimizing energy input. Integrating bioelectrocatalysis with carbon capture technologies [149] and multistep reaction pathways could further enhance efficiency by reducing the need for costly separation and purification steps. Ideally, a streamlined process using only CO_2_, protons, and electrons as inputs could produce final products directly. Such advancements would strengthen the potential of bioelectrocatalytic technologies to convert CO_2_ into valuable chemicals using renewable electricity, paving the way for a more sustainable chemical industry.

## Conflicts of Interest

The authors declare no conflicts of interest.

## Data Availability

Data sharing is not applicable to this article as no new data were created or analyzed in this study.
